# Differences in stakeholder-reported barriers and implementation strategies between counties with high, middle, and low HPV vaccine initiation rates: a mixed methods study

**DOI:** 10.1186/s43058-022-00341-y

**Published:** 2022-09-06

**Authors:** Stephanie A. S. Staras, Amanda L. Kastrinos, Easton N. Wollney, Shivani Desai, La Toya J. O’Neal, Versie Johnson-Mallard, Carma L. Bylund

**Affiliations:** 1 Department of Health Outcomes and Biomedical Informatics, College of Medicine, University of Florida, 2004 Mowry Road, Room 2238, Gainesville, FL 32610 USA; 2 The Institute for Child Health Policy, University of Florida, Gainesville, FL USA; 3 Department of Psychiatry & Behavioral Sciences, Memorial Sloan Kettering Cancer Center, New York, NY USA; 4 Department of Family, Youth, and Community Sciences, University of Florida, Gainesville, FL USA; 5 College of Nursing, University of Florida, Gainesville, FL USA

**Keywords:** Human papillomavirus vaccine, Expert Recommendations for Implementing Change, Stakeholder interviews, Mixed-methods

## Abstract

**Background:**

A greater understanding of the county-level differences in human papillomavirus (HPV) vaccination rates could aid targeting of interventions to reduce HPV-related cancer disparities.

**Methods:**

We conducted a mixed-methods study to compare the stakeholder-reported barriers and efforts to increase HPV vaccination rates between counties within the highest, middle, and lowest HPV vaccine initiation (receipt of the first dose) rates among 22 northern Florida counties. Between August 2018 and April 2019, we recruited stakeholders (*n* = 68) through purposeful and snowball sampling to identify potential participants who were most knowledgeable about the HPV vaccination activities within their county and would represent a variety of viewpoints to create a diverse picture of each county, and completed semi-structured interviews. County-level HPV vaccine initiation rates for 2018 were estimated from the Florida Department of Health’s immunization registry and population counts. Implementation strategies were categorized by level of importance and feasibility using the Expert Recommendations for Implementing Change (ERIC) taxonomy. We compared the barriers and implementation strategies for HPV vaccination between tercile groups of counties by HPV vaccine initiation rates: highest (18 stakeholders), middle (27 stakeholders), and lowest (23 stakeholders).

**Results:**

The majority of the 68 stakeholders were female (89.7%), non-Hispanic white (73.5%), and represented a variety of clinical and non-clinical occupations. The mentioned barriers represented five themes: healthcare access, clinician practices, community partnerships, targeted populations, and cultural barriers. Within themes, differences emerged between county terciles. Within healthcare access, the highest rate county stakeholders focused on transportation, lowest rate county stakeholders focused on lack of clinicians, and middle county stakeholders mentioned both. The number of ERIC quadrant I strategies, higher feasibility, and importance described decreased with the tercile for HPV vaccination: highest = 6, middle = 5, and lowest =3 strategies.

**Conclusions:**

The differing barriers and strategies between the highest, middle, and lowest vaccination rate counties suggest that a tailored and targeted effort within the lowest and middle counties to adopt strategies of the highest rate counties may reduce disparities.

Contributions to the literature

- Human papillomavirus (HPV) vaccination rates remain below national objectives in the USA with wide variations in the rates by geography; however, little is known about the local barriers and implementation strategies that attribute to these differences.
- The results of this study suggest that the Expert Recommendations for Implementing Change (ERIC) taxonomy was a useful tool to compare the implementation strategies on the importance and feasibility between the groups of high, middle, and low performers.
- Stakeholders reported a decreased number of high-importance implementation strategies ERIC quadrant I, by decreasing HPV vaccination rate tercile; shifting resources to higher-importance strategies may reduce vaccination disparities.

## Background

In the USA, human papillomavirus (HPV) vaccine initiation (i.e., ≥ 1 dose) and up-to-date (i.e., 2 doses if started the series < 15 years of age or 3 doses if started at ≥15 years or are immunocompromised) rates among 13- to 17-year-olds remain well below the national objectives (75% initiation and 59% up to date) [1, 2]. HPV vaccination is recommended for universal coverage at ages 11 to 12 years, although the vaccine can be given as young as age 9 and up to age 45 years [3]. Among the 50 states in the USA, Florida ranks 44th in HPV vaccine initiation (68%) and 41st for up-to-date (52%) among 13- to 17-year-olds [1]. Furthermore, the risk for HPV-related cancers is high in Florida (14.8 per 100,000 persons) as the state of Florida has the fifth highest rate of HPV-associated cancer rates among all 50 states in the USA [4]. Similar to other states [5–8], HPV vaccine initiation rates vary dramatically across Florida counties: 2018–2019 initiation rates among 13- to 17-year-olds by county ranged from 38 to 100% for females and 34 to 96% for males [9].

Social determinants of health, including rurality, race/ethnicity, poverty, and healthcare access, are associated with county-level differences in HPV vaccination rates [5, 9–11]. In addition, it is likely that differences in local barriers and current implementation strategies contribute to county variability in HPV vaccination. Increasing the understanding of the relationships between geographic HPV vaccination rates and barriers and/or implementation strategies could identify the best practices and, therefore, aid in the reduction of disparities.

HPV vaccine stakeholders (e.g., clinicians, parents, health department staff) have identified a variety of barriers to HPV vaccination [12–16]. The main identified barrier to HPV vaccination is parental lack of HPV vaccine awareness and limited knowledge about the vaccine, especially concerns about vaccine safety and limited understanding of the links between HPV and sexual activity or cancer [12–17]. Another prominent barrier identified by stakeholders is clinician discomfort with or lack of routine recommendations of the HPV vaccine [13, 16, 17]. While these barriers are consistently identified across stakeholder studies, it is unclear if stakeholder perceptions of barriers differ between areas with higher or lower HPV vaccination rates.

Across the USA, local vaccine stakeholders are enacting implementation strategies to increase HPV vaccination [13, 17, 18]. A common implementation strategy is addressing the lack of parental HPV vaccine awareness by distributing educational materials at clinics and community events [13, 17, 18]. Within educational material distribution, however, local practices are diverse [18]. For example, health departments across the five states of the Appalachia region provided parents with education information from different sources (Centers for Disease Control and Prevention, Merck Inc., or locally developed) [18]. It is reasonable to expect differences in implementation strategies to influence HPV vaccination rates, and one study in Texas suggested that locally tailored educational material increased regional vaccination rates when compared to neighboring counties [8].

To better understand the reasons for the county-level variation of HPV vaccination rates among 13- to 17-year-olds, we conducted a mixed-methods study examining the differences in stakeholders’ reports of county-level barriers to HPV vaccination and local implementation strategies between the highest, middle, and lowest HPV vaccine initiation rate counties. An increased understanding of the reasons behind these differences may identify promising implementation strategies for counties with lower HPV vaccination rates, thus aiding all counties in reaching the American Cancer Society’s goal of achieving 80% of 13-year-olds up to date for HPV vaccination by 2026 [2].

## Methods

### Study population

In 2018 and 2019, we conducted a mixed-methods environmental scan of the University of Florida Health Cancer Center (UFHCC) catchment area using the PRECEDE-PROCEED model as a structure for assessing the communities’ needs in order to develop tailored HPV vaccine initiatives [19]. The UFHCC catchment area was defined as the following 22 north central Florida counties: Alachua, Baker, Bradford, Citrus, Clay, Columbia, Dixie, Gadsden, Gilchrist, Hamilton, Jefferson, Lafayette, Leon, Levy, Madison, Marion, Putnam, Sumter, Suwannee, Taylor, Union, and Wakulla. For each county, we estimated HPV vaccine initiation rates; examined important, publicly available social determinants of health; and conducted telephone-based, semi-structured interviews with stakeholders.

### HPV vaccine initiation rates

We estimated county-level vaccinations with records of HPV vaccinations from the statewide Florida immunization information system, Florida State Health Online Tracking System (Florida SHOTS™), obtained from the Florida Department of Health. Florida SHOTS™ includes the immunization records from Florida county health departments and participating clinicians [20]. Using Florida SHOTS™ is useful because it is available at the county level and is mandatory for clinicians practicing at county health departments and participating in the federal Vaccines for Children program. As of 2019, 74% of Florida 11- to 17-year-olds had at least 2 immunizations recorded in Florida SHOTS™ [20]. Following our previously validated strategy [9], we estimated 2018 HPV vaccine initiation rates for each county by dividing the number of 13- to 17-year-olds receiving at least one dose of the HPV vaccine by 2018 by the total estimated population of 13- to 17-year-olds in 2018. We obtained the county-level population counts from publicly available data from the Florida Department of Health [21].

### Social determinants of health

Counties in the UFHCC catchment areas are mostly rural based on Florida Statutes Section 288.0650 (16/22 counties), a majority (74%) of the population is White, and 16% of the population lives beneath the national poverty line [22]. To incorporate county-level differences in social determinants of health, we included the four county-level characteristics that were associated with Florida county-level HPV vaccination among 11- to 12-year-olds in our previous analysis [11]. Among 27 publicly available county-level characteristics from the 2010 Census and the 2018 American Community Survey accounting for racial/cultural (e.g., race, immigration status), socioeconomic status (e.g., living below poverty level, education), and healthcare access (e.g., insurance status/type), HPV vaccination rates were associated with rurality [23, 24], family physicians per 100,000 residents [25], percentage uninsured [26], and percentage Medicaid-enrolled [25]. Due to the importance of race/ethnicity and poverty on HPV vaccination in the literature [5, 10], we also included the percentage of non-Hispanic white persons, of non-Hispanic black persons, and persons living below poverty [25, 27].

### Stakeholder interviews

We aimed to complete three stakeholder interviews for each county within the catchment area. We considered stakeholders to be anyone invested in increasing HPV vaccination including healthcare professionals, county extension agents from the UF Institute of Food and Agricultural Science Extension, community leaders, or parents. We used a purposive sampling approach to identify potential participants who were most knowledgeable about the HPV vaccination activities within their county and would represent a variety of viewpoints to create a diverse picture of each county [28]. Specifically, we reached out to known contacts, asked for recommendations from our community advisory board, and conducted Internet searches. As a secondary strategy, we used snowball sampling in which participants were invited to recommend two to three individuals whom would they thought would be interested in completing an interview.

Between August 2018 and April 2019, we attempted to contact potential participants up to four times, alternating between email and telephone calls. When a stakeholder expressed interest, a study staff member established a time to complete the interview via telephone. Before beginning the interview, the study staff consented participants under a Health Insurance Portability and Accountability Act waiver of documentation of consent. The study staff members that completed the interviews were masters-level trained in public health or related disciplines, were trained to the specific semi-structured interview script by a communication and qualitative expert (CLB), and had no prior relationships with the stakeholders. The first two interviews by each interviewer were reviewed by two authors (CLB and SAS), and feedback was provided to enhance the interviewers’ skills and study-specific probing. In total, 67 interviews of 68 stakeholders were conducted over the phone and audio-recorded with handheld devices. Upon completion of the interviews, participants were offered a $25 gift card. Recordings were immediately transferred to a secure server and uploaded via a Health Insurance Portability and Accountability secure site for transcription. Transcripts were created verbatim and deidentified by a third party. Coders received anonymized transcripts. All procedures were approved by the University of Florida Institutional Review Board.

### Stakeholder interview questions

Following the PRECEDE portion of the PRECEDE-PROCEED model [19], our semi-structured interviews included questions to assess the community factors influencing the initiation and completion of HPV vaccination. Social assessment questions assessed the priority of HPV vaccination, attitudes, goals to increase vaccination, main issues surrounding HPV vaccination within the county, and county resources. To assess the epidemiological factors, questions focused on which groups of people need the most support to start or complete the HPV vaccination series and the main motivating factors for adolescents to get the HPV vaccine. Lastly, implementation questions prompted stakeholders to discuss any current intervention efforts, barriers to the intervention/s, and their perception of the most important factors to improve HPV vaccination rates within their county.

### Analysis

For qualitative analysis, we started by identifying two major categories based on the interview script: [1] barriers and [2] implementation strategies. Each category focused on both initiating the vaccine and completing the vaccine series. We then conducted a thematic analysis of data within these categories using the constant comparative method [29]. We chose this method as it allowed us to approach the data inductively and iteratively using an interpretivist paradigm. First, two authors (CLB and ALK) became immersed in the data by reading the transcripts multiple times. Coders had extensive training and experience in qualitative research coding, and one (CLB) was familiar with the literature on HPV vaccination barriers and implementation strategies. We then identified the concepts within the text and assigned labels (codes) to those concepts. Codes were then grouped into categories using Owen’s criteria for thematic salience (recurrence, repetition, and forcefulness) [30]. Finally, we identified properties within these saturated themes, using them to develop a coding manual. To increase the comprehensiveness and trustworthiness of the analysis, the coding manual was verified by one author (SAS), an expert in HPV vaccination, and used to train a third coder (EW), trained in qualitative analyses, who validated previous analyses and helped complete the coding process.

After the coding process was complete, one author (SAS), an expert in HPV vaccination strategies and implementation science, and a trainee categorized the implementation strategies described by stakeholders into the 73 discrete implementation strategies as categorized by the Expert Recommendations for Implementing Change (ERIC) taxonomy [31, 32]. The trainee proposed implementation strategies, and then the trainee and author discussed the categorization until consensus was reached. The ERIC taxonomy was developed to consistently categorize and select implementation strategies [31, 33]. The ERIC taxonomy categorizes the implementation strategies into the four quadrants: higher feasibility and higher importance (quadrant I), higher feasibility and lower importance (quadrant II), lower feasibility and lower importance (quadrant III), and lower feasibility and higher importance (quadrant IV) [32].

After conducting the interviews, we decided to compare the stakeholder responses by county-level HPV vaccination rates. Thus, we divided the counties into terciles based on the 2018 HPV vaccine initiation rates. Dividing the 68 stakeholders interviewed into the highest, middle, and lowest terciles for HPV vaccination rates yielded 18 stakeholders from counties within the highest tercile (Alachua, Dixie, Gadsden, Hamilton, Leon, Levy, and Wakulla), 27 stakeholders from counties from the middle tercile (Bradford, Gilchrist, Levy, Marion, Putnam, Taylor, Sumter, and Suwanee), and 23 stakeholders were from counties in the lowest tercile (Baker, Citrus, Clay, Jefferson, Lafayette, Madison, and Union). Stakeholders representing multiple counties (*n* = 9) were grouped accordingly: three stakeholders in the highest tercile (2 representing two high counties and 1 representing two high counties and one middle county), five in the middle tercile (3 representing two middle counties and one high county and 2 representing one middle and one lowest where one spoke mainly of the middle county), and one in the lowest tercile representing 2 lowest counties. Due to the small number of stakeholders per county (*n* = 1 to 5) and our recruitment of diverse stakeholders, we did not assess the saturation within counties.

## Results

County-level HPV vaccine initiation rates among 13- to 17-year-olds in 2018 ranged from 44 to 62% in the lowest tercile, 63 to 70% in the middle tercile, and 71 to 82% in the highest tercile (Table 1). Based on a visual assessment of averages, compared to the lowest and middle terciles, the highest tercile had a slightly lower percentage of the population living in rural areas (51.9% highest versus 65.8% middle and 65.2% lowest), a greater density of family physicians per 100,000 (18.4 highest versus 9.2 middle and 10.6 lowest), a lower percentage of non-Hispanic whites (68.1% highest versus 81.9% middle and 75.6% lowest), and a higher percentage of non-Hispanic African Americans (25.9% highest versus 13.2% middle and 19.9% lowest).

**Table 1:** County-level HPV vaccine initiation and select population demographic factors

	HPV vaccine initiation^**a**^	% living in rural areas	Family physicians per 100,000	Uninsured, %	Medicaid insured %	Non-Hispanic White, %	Non-Hispanic African American, %	Below poverty, %
Highest tercile								
Gadsden	82.0%	65.4%	8.3	16.3%	25.5%	35.9%	56.0%	24.6%
Alachua	79.6%	21.2%	49.7	11.9%	15.5%	69.6%	20.3%	21.8%
Leon	78.4%	12.3%	42.7	10.8%	13.9%	63.0%	30.3%	20.4%
Hamilton	72.8%	63.5%	0	15.1%	24.9%	59.8%	34.5%	29.5%
Dixie	72.1%	77.0%	6	15.8%	27.6%	88.8%	8.4%	23.1%
Wakulla	71.1%	61.7%	9.3	12.4%	12.9%	82.0%	14.5%	11.5%
Columbia	71.0%	62.1%	12.9	12.4%	25.1%	77.9%	17.5%	17.4%
Average	75.3%	51.9%	18.4	13.5%	20.8%	68.1%	25.9%	21.2%
Middle tercile								
Marion	69.7%	31.0%	16.3	15.4%	21.1%	81.0%	12.3%	16.6%
Gilchrist	69.2%	83.9%	11.4	15.5%	21.2%	90.9%	5.3%	17.9%
Suwanee	67.7%	83.2%	4.4	17.2%	25.3%	82.5%	11.4%	17.8%
Putnam	66.7%	56.2%	8.2	17.2%	29.8%	77.3%	16.2%	24.8%
Taylor	64.7%	69.3%	4.5	14.9%	23.2%	75.2%	20.7%	19.8%
Bradford	64.7%	75.5%	10.7	13.3%	21%	76.4%	20.4%	20.3%
Levy	64.1%	92.1%	4.8	18%	23.3%	85.5%	9.4%	20.7%
Sumter	62.4%	35.0%	13.5	11.9%	8.3%	86.6%	9.7%	8.8%
Average	66.2%	65.8%	9.2	15.4%	21.7%	81.9%	13.2%	18.3%
Lowest tercile								
Clay	62.3%	15.0%	20.1	11.4%	14.3%	81.8%	9.9%	10.6%
Lafayette	59.3%	100%	0	19.8%	16.4%	77.4%	15.9%	20.1%
Union	56.5%	67.5%	6.3	12.7%	16.3%	75.0%	22.2%	22.0%
Jefferson	50.4%	100%	13.6	13.6%	18.3%	60.4%	36.2%	14.1%
Citrus	48.6%	34.5%	15.2	15.4%	18.3%	93.0%	2.8%	16.7%
Baker	45.8%	59.5%	3.6	12%	21.5%	83.7%	13.6%	14.6%
Madison	43.7%	80.0%	15.5	14.2%	24.2%	57.6%	38.8%	28.2%
Average	52.4%	65.2%	10.6	14.2%	18.5%	75.6%	19.9%	18.0%

^a^Among 13- to 17-year-olds in 2018

The majority of the stakeholders were female (89.7%), non-Hispanic white (73.5%), and represented a variety of clinical and non-clinical occupations (Table 2). Overall, the demographic characteristics between stakeholders in the highest, middle, and lowest HPV vaccine initiation rate counties were similar. Some differences were visually apparent: stakeholders in the lowest vaccine initiation counties were more likely to be non-White race/ethnicity and not from private clinical practice, while stakeholders in the highest vaccine initiation rate counties were older and did not include public school nursing staff.

**Table 2:** Characteristics of stakeholders from counties with high, middle, and low vaccination rates

	Stakeholders from counties with high rates, *N* (%)	Stakeholders from counties with middle rates, *N* (%)	Stakeholders from counties with low rates, *N* (%)
Total	18	27	23
Gender			
Female	15 (83%)	25 (93%)	21 (91%)
Male	3 (17%)	2 (7%)	2 (9%)
Race/ethnicity			
Non-Hispanic White	15 (83%)	21 (78%)	14 (61%)
African-American	2 (11%)	4 (15%)	6 (26%)
Asian	1 (6%)	1 (4%)	0 (0%)
Hispanic	0 (0%)	1 (4%)	1 (4%)
Multiracial	0 (0%)	0 (0%)	2 (9%)
Age			
24–35	1 (6%)	6 (22%)	8 (35%)
36–50	8 (44%)	9 (33%)	7 (30%)
50+	9 (50%)	1 (45%)	8 (35%)
Occupation			
Clinician			
Department of Health	5 (28%)	9 (33%)	5 (22%)
Private practice	5 (28%)	3 (11%)	1 (4%)
Federally qualified health center	1 (5.5%)	0 (0%)	0 (0%)
Nonclinical staff in clinical or public health settings			
Department of Health coordinators and educators	2 (11%)	3 (11%)	4 (17.5%)
Private practice office manager or vaccine coordinator	2 (11%)	5 (19%)	3 (13%)
Public school nursing staff	0 (0%)	3 (11%)	3 (13%)
Other health professionals^a^	2 (11%)	3 (11%)	4 (17.5%)
Other^b^	1 (5.5%)	1 (4%)	3 (13%)

^a^Other health professionals includes University of Florida Institute of Food and Agricultural Science county Extension agents and Area Health Education Centers

^b^Other includes a reverend, high school mental health counselor, a high school teacher, a National Cervical Cancer Coalition member, the American Cancer Society staff member, and advocacy group director

Stakeholders from all settings, highest, middle, and lowest HPV vaccine initiation rate counties, mentioned barriers within five saturated themes (healthcare access, clinician practices, community partnerships, limited knowledge, and cultural barriers). Differences emerged within themes between the highest, middle, and lowest counties (Table 3). The number 1 barrier suggested by stakeholders was the lack of healthcare access available for HPV vaccination. When speaking about healthcare access, however, stakeholders from the highest rate counties focused on a lack of transportation to healthcare, stakeholders from the lowest rate counties focused on the limited number of local primary care physicians, and stakeholders from the middle rate counties mentioned both transportation and limited local physicians.

**Table 3:** Stakeholder-reported barriers to county-level HPV vaccine initiation

Thematic category	County-level vaccine rate	Emphasis	Representative quotes
**Healthcare access**	**Highest**	Transportation	*I know the transportation can be an issue in our community sometimes for people even that live locally to get to the health department.*—Healthcare provider
	**Middle**	Transportation and limited healthcare professionals	*There’s already transportation issues, and time issues where if it’s a single parent who has to take two buses to the doctor.*—Area Health Education Center staff *We are limited in our – the amount of pediatricians that we have.*—DOH clinical staff
	**Lowest**	Limited healthcare professionals	*We have limited numbers of physicians there [in the area] … maybe five family physicians at the most.*—DOH nonclinical staff
**Clinician practices**	**Highest**	Recommendation variability	*A lot of the pediatricians they don’t want to give the vaccine. I was told last year, she doesn’t need it now and she was – she was 10 last year. So, she could have gotten the vaccine but even the pediatrician was like, “Oh no, you have to wait.”*—DOH nonclinical staff
	**Middle**	Refer to health department	*It*’*s so expensive. I don’t know that there’s very many [pediatricians] that keep it in stock … most of them will send them here.*—DOH clinical staff
	**Lowest**	Refer to health department	*Pediatricians do not offer HPV vaccine and just refer patients to health department.*—Health educator
**Community partnerships**	**Highest**	School	*Definitely within the school system and county officials, we have some difficulties there, trying to promote anything that is outside of an abstinence-only model. So, we have difficulty promoting in that way or having the support to promote, you know, on a larger scale.*—Healthcare provider
	**Middle**	School	*We don’t really do vaccines at schools because the parents aren’t very receptive to that here. … We can offer it, but it don’t usually happen because of the parents and school board.*—DOH clinical staff
	**Lowest**	Church	*They’re [the church] not going to talk about anything that’s seen as sexual, and the HPV is really seen as a sexual disease when it’s not just about sex.*—UF Institute of Food and Agricultural Science Extension agent
**Limited knowledge**	**Highest**	Healthcare provider	*We believe that the providers needed a bit more education about the HPV vaccine before they were more comfortable with promoting it.*—Health educator
	**Middle**	Parents	*Some people are just afraid because they’re unfamiliar, because they didn’t get it when they were a child*.—DOH nonclinical staff
	**Lowest**	Parents/community	*[In the community] I would just say lack of knowledge and the understanding of it.*—Public school faculty/staff
**Cultural barriers**	**Highest**	Vaccine exemption	*Oh, actually, there is one thing I think is important for you to know. Our county is, I believe, the highest county in the state for religious exemption, and that’s a huge barrier for us to, you know, immunize kids, because they’re not only refusing HPV.*—Healthcare provider
	**Middle**	Vaccine exemption	*We have a high percentage of religious exemptions, and it’s not from religion, it’s from personal exemptions. It’s a false messaging from vaccines in general. … [Parents] are leery of any vaccine.*—DOH clinical staff
	**Lowest**	Religious beliefs	*I think as far as barriers go, one, the religious thing. This is a very religious community and HPV’s not the only thing that we struggle with educating the community on.*—Public school faculty/staff

Stakeholders from all types of counties mentioned clinician practices as a barrier. Yet, the highest rate county stakeholders focused on the quality and strengths of clinician recommendations, and the middle and lowest rate county stakeholders focused on clinicians referring patients to the health department for vaccinations instead of offering the vaccine on site. Similarly, stakeholders from all county terciles mentioned difficulties with community partnerships: highest and middle rate county stakeholders focused on difficulties partnering with schools and the lowest rate county stakeholders discussed difficulties partnering with churches. All groups discussed limited knowledge as a barrier to HPV vaccine; however, the highest rate county stakeholders focused the discussion on healthcare providers, and the middle and lowest rate county stakeholders focused on parents in the community. Finally, cultural barriers were a focus of all groups with the highest and middle rate county stakeholders focusing on vaccine exemptions and the lowest rate county stakeholders focusing on religious beliefs.

Overall, the number of implementation strategies described by stakeholders decreased by HPV vaccination tercile: 20 in the highest, 19 in the middle, and 13 in the lowest rate counties (Table 4). Similarly, within ERIC quadrant I, the strategies considered of higher feasibility and higher importance, the number of strategies described decreased with the tercile for HPV vaccination: 6 in the highest, 5 in the middle, and 3 in the lowest rate counties. Stakeholders from the highest rate counties described strategies that directly targeted the main influencers of HPV vaccination, parents and clinicians, whereas stakeholders from the lowest rate counties described strategies that focused on training school nurses to educate students. Stakeholders from the middle rate counties mentioned both parent- and school-targeted strategies. Stakeholders from both the highest and middle rate counties mentioned following the Center for Disease Control and Prevention’s recommended strategy of presenting the three adolescent vaccines together on the same day in the same way. One stakeholder from the highest rate county mentioned training clinicians in this strategy by stating:

**Table 4:** Interventions categorized by discrete implementation strategies in the expert recommendations for implementing change (ERIC)

Discrete implementation strategies	Quadrant	Highest rates	Middle rates	Lowest rates
**Facilitate relay of clinical data to clinicians**	**Higher feasibility and higher importance (I)**			
Keep records/check state immunization registry		**X**	**X**	**X**
**Identify and prepare champions**				
School nurses as HPV vaccine champions		**X**	**X**	**X**
Survivor of HPV-related cancer		**X**		
**Conduct ongoing training**				
Train pediatricians and clinic staff to communicate with parents		**X**	**X**	
Training school nurses to educate students			**X**	**X**
**Distribute educational materials to stakeholders**				
Utilize local library to pass out information about the vaccine		**X**		
Sending education materials to local vaccine health care clinicians		**X**	**X**	
**Prepare patients/consumers to be active participants**	**Lower feasibility and higher importance (IV)**			
Deliver information to parents via schools		**X**	**X**	**X**
Health educator at school talks to students		**X**		**X**
HPV discussed in public school sex education		**X**		
Digital clinic waiting room sign information		**X**	**X**	
School nurses focus vaccine messaging on cancer prevention			**X**	**X**
**Intervene with patients/consumers to enhance uptake and adherence**				
Health fairs		**X**	**X**	**X**
Promotional items and financial incentives		**X**	**X**	**X**
Dentists educate patients on the links between HPV and oral cancer		**X**	**X**	**X**
Reminder text message to parents		**X**	**X**	
School events with vaccines available		**X**	**X**	
Vaccines at family planning clinics		**X**	**X**	
Offering bus passes to patients		**X**	**X**	
Discuss vaccine at all health appointments			**X**	**X**
**Alter patient/consumer fees**	**Lower feasibility and lower importance (III)**			
Discount, sliding scale, or free		**X**	**X**	**X**
**Use of mass media**				
National TV advertisements from Merck		**X**	**X**	**X**
Local advertising campaigns (e.g., newspaper, radio advertisements)		**X**	**X**	**X**

We have done several trainings with providers in teaching them about the ‘Same Day, Same Way,’ helping them to dispel myths, providing them with education about different cancers associated with HPV.

In contrast, a representative stakeholder from the lowest vaccination rate counties mentioned training focused on using school nurses to educate students about the vaccine, stating:

education provided, again, by the health department for the school nurse liaison as well as the school nurses.

Similarly, within quadrant IV strategies, considered lower feasibility and higher importance, stakeholders from the highest and middle rate counties mentioned the most strategies (11 strategies) and stakeholders from the lowest rate counties mentioned the fewest (7 strategies). While stakeholders from all three tercile rate counties mentioned using schools to deliver information to parents, only the highest rate county stakeholders mentioned incorporating HPV in public school sex education. Stakeholders from middle and lower rate counties mentioned that school nurses focus on cancer prevention when discussing the HPV vaccine.

Stakeholders from all tercile vaccination rate counties mentioned strategies that directly intervened with parents including participating at health fairs, offering incentives, and dentists discussing the links between HPV and oral cancer. Stakeholders from higher and middle rate counties mentioned using strategies to directly address barriers including sending reminder text messages to parents, offering vaccines at schools or family planning clinics, and offering bus passes. For example, one healthcare provider from a county with a higher vaccination rate described a strategy at schools that provided education and administered the vaccine:

There is a school kind of firmly planted in that area where, you know, most of those particular children actually attend, and we did partner with them and offer vaccines at their site.

In contrast, a stakeholder from the lowest vaccine rate county described a strategy at schools teaching students about the vaccine:

So, you know, again, it’s not like a scared straight type of deal, but it’s kind of similar. You have to show those graphics, so they can actually see that there is a potential for them to get that if they don’t get vaccinated. So, they can actually become their own advocates and tell their parents they do want that.

Finally, while some stakeholders from middle tercile counties were able to offer vaccines at schools, most mentioned that schools in their area were resistant to the idea. For example, one stakeholder said:

There are some counties we’ve heard where they’re actually doing the vaccines in school. … We haven’t had a whole lot of success in communicating with schools to do that.

Stakeholders from all tercile vaccination rate counties did not mention quadrant II strategies, higher feasibility, and lower importance and described a similar number and type of quadrant III strategies considered lower feasibility and lower importance.

## Discussion

Important differences in barriers and implementation strategies emerged between northern Florida counties with the highest, middle, and lowest HPV vaccine initiation rates, while stakeholders reported barriers focused on similar themes to each other and prior literature [12–17, 34], stakeholders in counties with the highest, middle, and lowest vaccine initiation rates emphasized different constructs within themes. Differences also emerged between the highest, middle, and lowest vaccination rate counties’ implementation strategies. For example, stakeholders from the highest rate counties were more likely than stakeholders from middle and lower rate counties to report strategies of higher importance. Increasing the use of higher importance strategies within lower and middle vaccination rate counties may help reduce the county-level disparity in HPV vaccination.

Consistent with previous research [12–17, 34], stakeholders in all terciles of HPV vaccination counties identified parents’ knowledge and clinicians’ practices as the main barriers to HPV vaccination. Our identification of healthcare access as the main barrier to HPV vaccination within north-central Florida suggests that it may be a more salient barrier for rural populations, which aligns with previous research as it was mentioned only in one of the previous HPV vaccine stakeholder studies for the remote counties only [12]. Finally, our lowest rate county stakeholders’ comments expanded prior findings of churches as promising community partners by emphasizing the difficulties of engaging churches and religious communities around the HPV vaccine [12, 35].

Differences between higher, middle, and lower vaccination rate counties add evidence to the possibility of local implementation strategies influencing community vaccination rates [8, 36]. For example, contrasting the findings of prior stakeholder studies and our higher vaccination rate counties that focused on the quality of clinician recommendations [12, 16, 17], stakeholders in middle and lower vaccination rate counties highlighted clinician practices of referring patients to the health department for vaccinations. While clinician referral of vaccinations to health departments is a known vaccination barrier [37], physicians, especially rural physicians, report referral of vaccinations due to inadequate reimbursement, parent request, and storage and stocking difficulties [38]. Potential interventions to address this barrier include helping clinicians enroll in Vaccines for Children or providing alternative vaccination sites such as pharmacies or mobile vaccination clinics [3, 13, 39–41]. Mobile clinics may be more feasible solutions in the middle and lowest vaccination rate counties as the difficulty maintaining vaccine stock is likely exacerbated by the greater percentage of the population in these counties living in rural areas and the limited number of family physicians [23–25].

Our application of the ERIC framework to the qualitative interviews from stakeholders strengthens and expands the use of the ERIC framework as a strategy to evaluate the differences in implementation strategies between the high- and low-performing groups. The ERIC strategy has been used to quantitively divide the strategies reported in the focus groups [42]. One study compared the ERIC strategies between sites by providing a list of ERIC strategies and asking sites to endorse strategies they use [43]. Consistent with our findings, this study found sites treating more patients for hepatitis C virus were more likely to endorse using highly feasible and higher importance ERIC strategies than sites treating fewer patients [43].

Importantly for the promotion of HPV vaccination, the identified differences in ERIC framework implementation strategy importance between high, middle, and low tercile vaccination counties suggests that implementing higher importance strategies in lower tercile counties may help resolve HPV vaccination disparities. In particular, only stakeholders from the highest and middle tercile HPV vaccine initiation counties described using well-established, evidence-based implementation strategies classified as higher feasibility and higher importance, including reminder-recall messages for parents and training clinicians to strengthen their HPV vaccine recommendations [44–47]. Stakeholders from the highest and middle rate counties also described addressing vaccine access by employing multiple evidence-based, lower feasibility, and higher importance strategies to offer alternative vaccination sites (i.e., schools and family planning clinics) [48–50].

Our study includes three important limitations. First, the collected data is a cross-sectional snapshot of practices and vaccination rates in the counties. Thus, we cannot evaluate the cause-and-effect relationship between implementation strategies and vaccination rates. Second, all data were collected at the county level, and important differences in HPV vaccination, barriers, and implementation strategies within counties may have been overlooked. Third, there are important demographic differences (e.g., rural population and number of family physicians) between county terciles that likely affect HPV vaccination rates in conjunction with their implementation practices. For example, it may be more difficult to counteract HPV vaccine misconceptions in the more rural areas typical of the middle and lowest tercile due to the limited healthcare providers, greater parent vaccine hesitancy, and transportation difficulties [51–54]. Additionally, while consistent with another county-level study showing higher vaccination coverage in counties with a greater percentage of non-Hispanic African Americans [5], our interviews did not allow us to untangle the interactions between responsiveness to vaccine promotion program implementation and possible trust and healthcare access issues influenced by poverty and race/ethnicity [55, 56].

Our study includes three important strengths. First, expanding other stakeholder studies of HPV vaccination [12–16], we combined stakeholder interviews with HPV vaccination data to compare the barriers and implementation strategies between high, middle, and low vaccination rate counties. Second, we included a broad range of community stakeholders to create a more comprehensive view of the county’s culture. Third, we enhanced our needs assessment by applying the established ERIC implementation taxonomy to compare the local implementation strategies targeting HPV vaccine interventions. This comparison allowed us to identify specific strategies that were occurring in higher and not lower tercile counties.

## Conclusions

Comparing stakeholder opinions about barriers and implementation strategies between the highest, middle, and lowest tercile counties for HPV vaccine initiation revealed important differences. The ERIC implementation taxonomy was particularly useful in classifying the importance and feasibility of strategies used by the higher, middle, and lower tercile counties. The study provides an example of how the ERIC implementation taxonomy can be used to compare implementation strategies between the groups. Taking the unique barriers of limited healthcare professionals and religious concerns into account, lower and some middle tercile counties could be encouraged to adopt implementation strategies of higher importance such as reminder/recall, providing alternative locations for vaccinations, training clinicians in recommendation strategies, and addressing barriers to clinician participation in Vaccines for Children. Achieving equity for HPV vaccination across Florida will require tailored and targeted efforts within lower vaccination rate counties.

## Data Availability

The datasets used and/or analyzed during the current study are available from Dr. Stephanie Staras upon reasonable request.

## Abbreviations

HPV: Human papillomavirus
ERIC: Expert Recommendations for Implementing Change
UFHCC: University of Florida Health Cancer Center Cancer
